# Supplementary material for: Universal method to extract the average electron spin relaxation in organic semiconductors from muonium ALC resonances
Source: Sci Rep. 2025 Nov 27;15:42301. doi: 10.1038/s41598-025-25045-w (PMC12660779; doi:10.1038/s41598-025-25045-w)
Supplement: Supplementary file 1 — Supplementary Information. [file 41598_2025_25045_MOESM1_ESM.pdf]

1 Appendix A: a typical example of the Python code used for generating our model

```
import numpy as np
from quantumtabletools import RunModelledSystem

Rlist=[]
for A in range(400,451,5): # the HFCC A
    D = 10 # the HFCC D
    E = 10 # the HFCC E
    eSR = 0 # eSR
    # setup of Quantum table
    Tab={}
    Tab['spins']=['Mu','e']
    Tab['dynamic']=[1]
    Tab['a(@0,Mu)']=[A,D,E]
    Tab['relax(@0,e)']=[eSR]
    Tab['lfuniform']=[25]
    Tab['loop0par']='bmag'
    # field range and steps
    Tab['loop0range']=[0,2.6,261]
    Tab['measure']=['integral']
    #run Quantum simulation and read back output
    xaxes,ydata,edata = RunModelledSystem(Tab)
    Rlist.append([A, D, E, eSR,
                 ydata[0].tolist()])

#save output
OutputfileName='Mue_loopADEeSR_Simulation.txt'
f=open(OutputfileName,'w')
for i in range(len(Rlist)):
    s=map(lambda x: "{:.5f}".format(x),
          Rlist[i][4])
    print(Rlist[i][0],Rlist[i][1],Rlist[i][2],
           Rlist[i][3],*s, file=f)
f.close()
```

You will obtain the LF dependence of the muon's polarization, $P(B)$, as a function of eSR, the isotropic (A) and anisotropic (D, E) hyperfine coupling constants. To calculate the integrated polarization loss ratio, it is necessary to determine the full field range of the ALC resonance from Bmin to Bmax, the minimum and maximum fields chosen to minimize systematic errors. Specifically, reducing Bmin (unless the ALC resonance falls within the repolarization field range) or increasing Bmax would not alter the integrated polarization loss with 0 eSR beyond a reasonable level of precision.

2 Appendix B: the verification of the robustness

```
# import numpy and matplotlib
import matplotlib.pyplot as plt
import numpy as np

from quantumtabletools import RunModelledSystem

# ALC modelling
def QuantumIntegralALC(A,D,E,ESR,Bmin,Bmax,Npts=200,ave=25,plotraw=False):
    table={
        "spins":("Mu","e"),
        "dynamic":(1,),
        "a(Mu)":(A,D,E),
        "relax(e)":(ESR,),
        "measure":("integral",),
        "loop0par":"bmag",
        "loop0range":(Bmin,Bmax,Npts),
        "lfuniform":(ave,),
        }
    x,y,e=RunModelledSystem(table)
    print ("done",D,E,ESR)
    if plotraw:
        return x[0],y[0]
    else:
        return np.sum(1-y)

# example of use
A=700
Bmin=2.2
Bmax=2.9
DEset=((10,10),(10,5),(10,0),(20,20),(20,10),(20,5),(10,20),(5,10))
ESRrange=np.linspace(start=0,stop=5,num=51)

# plot one of the ALC spectra to check field range
fig,ax=plt.subplots()
x,y=QuantumIntegralALC(A,DEset[-1][0],DEset[-1][1],ESRrange[0],Bmin,Bmax,plotraw=True)
ax.plot(x,y,"-")
plt.show()

# now integrate them for different E,D and scan ESR
fig,ax=plt.subplots()
for (D,E) in DEset:
    y=np.zeros([len(ESRrange)])
    for i,ESR in enumerate(ESRrange):
        y[i]=QuantumIntegralALC(A,D,E,ESR,Bmin,Bmax)
```

```
    y=y/y[0] # normalise to ESR=0
    ax.plot(ESRrange,y,label="D={} E={}".format(D,E))
ax.legend()
plt.show()
```

This Python code is used to verify the robustness of R_{IPL} with different EPR by changing parameters, such as the HFCCs (A, D, E) and eSR and the field range from Bmin to Bmax in the code.
